# On the Analysis of the Blood and Urine, in Health and Disease; and on the Treatment of Urinary Diseases

**Published:** 1846-07

**Authors:** 


### ART. XXIV.

*On the Analysis of the Blood and Urine, in Health and Disease; and on the Treatment of Urinary Diseases.* By G. OWEN REES, M.D., F.R.S., F.G.S., &c. Second edition. *London,* 1845. 8vo, pp. 218. With a plate.

WE have too long delayed noticing this very useful treatise, which may almost claim to be considered as a new work, rather than as a new edition of an older one, so great is the amount of alteration and addition it has received. These are in part occasioned by the advances which the science of animal chemistry has made since the first edition was published; but they partly consist also of the introduction of an essay on the treatment of urinary diseases, which nearly equals the original treatise in bulk. It is not the author's desire that his work should be regarded as an elaborated survey of the blood and urine in all their relations, but merely as a guide to proximate animal analysis, and to the practical application of the results of such analyses. His object has been throughout, he states in his preface, "simply to supply to the medical practitioner the means of analysing blood and urine, and thus to assist him to comprehend and appreciate with more defined ideas the experiments of those animal chemists whose results may have reference to the important subject of humoral pathology," A description of the microscopical appearances of urinary deposits, with illustrations, has also been added.

We are well assured that the analytical portion of this treatise is of peculiar value to the medical practitioner, supplying him with all the information he can require for ordinary purposes, without distracting his attention and burdening his memory with an extensive code of directions, which have reference to substances of rare occurrence, and which are consequently adapted for the exclusive benefit of those who make organic chemistry their special object of pursuit. We do not hesitate to say, that by following out the course which Dr. Rees has thus cleared, any medical man possessed of but ordinary skill in manipulation, at the expense of a little time and attention, may render himself so far conversant with the processes described as to be able to perform them all, as occasion may arise, in those intervals which occur in the professional avocations of almost every one; thereby securing a greatly-increased accuracy of diagnosis in a large and most important class of complaints, and also adding in no trifling degree to his power of discerning the true value of the researches of others, and of applying their results.

In regard to the practical portion of the work, we think Dr. Rees would have done wisely in not connecting this essay with a treatise which had acquired for itself the reputation of giving almost everything that it is desirable for the practitioner to know, on the subject which it professes to discuss; for its chief fault is its meagreness of detail, both in regard to the symptomatology of the diseases of which it treats, to the constitutional states with which they are connected, and to the treatment which will be most appropriate under different conditions. It appears to us that Dr. Rees underrates the influence of diet upon lithic-acid deposits, and in diabetes; and that he neglects an important principle in the treatment of the phosphatic deposits. Doubtless it is not in every case that these dietetic principles can be rigidly carried out, which the pathology of the two former of these disordered conditions would dictate; but where the system *can* accommodate itself to them, we feel assured, from evidence we have been at much pains to collect, that great relief may be given, and, in certain cases, even a permanent cure effected, by these means alone. Dr. Rees does not feel disposed to assent to the opinion of those who would connect the phosphatic deposits and alkaline urine with disorder of the nervous system; and he considers that, in by far the largest proportion of cases, the alkaline character is only acquired by the urine, after it has left the kidneys, in consequence of an unduly irritable state of the mucous membrane of the urinary passages, occasioning an excessive secretion of alkaline mucus. As a test of the correctness of this view, he adduces the benefit derived from the use of alkaline remedies in rendering the urine acid, and relieving the other symptoms; his idea of their *modus operandi* being, that they render the urine less acid and irritating at the time of its passage from the kidney, and consequently remove the cause for the undue secretion of mucus. Without denying that such may be the true pathology of certain forms of phosphatic deposit, we would take this opportunity of stating, that the result of many inquiries made subsequently to our review of Dr. Golding Bird's work, has fully confirmed the opinion we then expressed,—namely, that any excessive *waste* of the nervous system is a direct cause of phosphatic deposits in the urine. And we understand that the researches of Dr. Bence Jones, lately communicated to the Royal Society, and shortly (we hope) to be published, afford much additional evidence to the same effect.

